# Comparison of the possible histopathological changes of the rat neonatal cerebellum induced by toxic and nontoxic doses of biological silver nanoparticles with chemical silver nanoparticles

**DOI:** 10.1002/brb3.2319

**Published:** 2021-08-01

**Authors:** Hanieh Alsadat Mirkatuli, Mohammadhasan Baghbani, Behrooz Yahyaei

**Affiliations:** ^1^ Department of Medical Sciences, Shahrood Branch Islamic Azad University Shahrood Iran; ^2^ Department of Medical Sciences, Mashhad Branch Islamic Azad University Mashhad Iran; ^3^ Department of Medical Sciences, Biological Nanoparticles in Medicine Research Center, Shahrood Branch Islamic Azad University Shahrood Iran

**Keywords:** biological silver nanoparticles, cerebellum, chemical silver nanoparticles, histopathology, neonatal rat

## Abstract

**Introduction:**

Today, due to the increasing application of silver nanoparticles in medical products, it is necessary to pay attention to the user's safety. There are three methods, namely, chemical, physical, and biological, used for the production of nanoparticles. Although the first two methods might introduce health hazards, the latter is hypothetically safe. In this study, we examined the histopathological changes in the cerebellum of neonatal Wistar rats induced by injection of toxic and nontoxic doses of silver nanoparticles, which were produced by green synthetic method and were compared with chemical silver nanoparticles.

**Methods:**

This study was a laboratory interventional study performed on 25 Wistar rats in the Animal Laboratory of Islamic Azad University of Shahrood. These rats were divided into five groups of the control group, the group with nonpoisonous injection of chemical nanoparticles, the group with nonpoisonous injection of biological nanoparticles, the group with injection of poisonous chemical nanoparticles, and the group with injection of poisonous biological nanoparticles. The rats were impregnated by the males of the same race and the cerebellum of their offspring was studied after birth.

**Results:**

We found that the injection of nonpoisonous chemical nanoparticles caused hyperemia, inappropriate size, and dark cytoplasm in some Purkinje cells. Also, injection of poisonous chemical nanoparticles caused hyperemia and cellular dispersion in the molecular layer, caused abnormal shapes, and reduced the number of cells in Purkinje cells. However, injection of poisonous and nonpoisonous biological nanoparticles did not alter cerebellum cells nor did it cause any inflammation or hyperemia.

**Conclusion:**

In contrast with chemical nanoparticles, biological nanoparticles have less significant effect on the cerebellum cells.

## Abstract 2

Examined the histopathological changes in the cerebellum of neonatal wistar rats. Silver nanoparticles which are produced by green synthetic method. In contrast with chemical nanoparticles, biochemical nanoparticles have less significant effect on the cerebellum cells.

## 1 INTRODUCTION

Nowadays, silver nanoparticles are one of the most widely used materials in high‐consumption products, in the form of dispersed or solid particles with a size in the range of 10–100 nm. Studies show that silver nanoparticles with different cytotoxic and immunotoxic effects, sizes, and concentrations have different distribution and effects on body tissues (Trop et al., 2014; Wijnhoven et al., 2018).

Silver compounds with antibacterial and antifungal features are used in different ranges in medical applications, including clinical applications and withdrawal drugs, magnetic resonance imaging, and treatment of diseases. Recently, they have been widely used for drug delivery, gene delivery, cancer treatment, biosensors, photothermal therapy, and imaging techniques.

Note that it is also widely known as a carrier system for therapeutic molecules with the primary goal of improving the therapeutic effect and reducing side effects (Ahmadi & Kordestany, 2011; Guan et al., 2013; He et al., 2017). Although nanoparticles are known as safe materials, there are different reports about the toxic effects of the used nanoparticles in vivo. There are three different techniques for nanoparticles production, namely, biological, physical, and chemical methods (Pourali et al., 2013). The chemical method of nanoparticles production is fast and easy but toxic elements on the nanoparticles’ surface are of environmental concern. The physical method is time‐consuming and the produced nanoparticles might not be uniform in size. The biological method, also called green, uses microorganisms and plants for nanoparticles production (Pourali et al., 2017). This method of production is environmentally friendly and inexpensive. It is reported that some types of fungal and bacterial strains reduce the ions imposed to the culture media, and by converting them to the nanoparticle forms, the toxicity of materials will lessen. This reduction occurs through nonenzymatic and enzymatic ways. In the former, the enzymes which are present in or out of the microorganism's cell act on the toxic ions and reduce them to nanoparticles, while in the nonenzymatic way, some microbially secreted extra cellular elements, such as the active groups of the proteins and polysaccharides, are responsible for the bioproduction of nanoparticles (Pourali et al., 2014). Among different types of metal nanoparticles produced by microorganisms are gold (Fayaz et al., 2011), silver, silica, zirconia, cadmium, and titanium (Bahrulolum et al., 2021). The application of these nanoparticles in medicine is based on the physical and chemical requirement, such as drug delivery(Sivanesan et al., 2021), antimicrobial agents (Patra & Beak, 2017), and medical imaging (Kethirabalan & Gurusamy, 2013 ). It has been reported that silver nanoaprticles (AgNPs) demonstrate optimum antimicrobial properties so they are widely used in medicine.

The central nervous system is an organ sensitive to any changes during the fetal period and is protected by a blood–brain barrier (BBB). The BBB contains a layer of endothelial cells and astrocytes that prevent the transport of various particles, such as nanoparticles and drugs. The cerebellum, the second largest part of the brain, is a useful and ideal example for studying the growth of neurons, because at each stage of development, distinct morphology and specific histological features can be identified with different cell types unique to each period (Prakash et al., 2017).

Nanoparticles have potential applications, which include the promotion and activation of neuronal cell differentiation as reported in both in vitro and in vivo models. Nanoparticles can also reverse the neurologic impairments in the animal models of neurologic disorders, such as brain ischemia and Parkinson's and Alzheimer's diseases. Research has shown that nanoparticles promote neuronal differentiation and enhance neuronal survival and neuronal growth and maturation. However, there are few nanoparticles that do not promote neuronal differentiation and cause neuronal damage or neurotoxicity. Most of the current data are based on morphologic, anatomical, and behavioral parameters, and we do not still know molecular mechanisms behind nanoparticle mechanism of action on neurons (Khan et al., 2018). The nervous tissue is a particularly sensitive target, because its cellular components (mainly neurons and glial cells) are tightly regulated and metabolically exigent biological entities (Lovisolo et al., 2018). There are some studies on the accumulation of silver nanoparticles (SNPs) in the kidney and liver of the experimental animals and their transmission through the BBB and placenta that may affect the function of these organs. For example, it was shown that the 25‐nm‐sized SNPs that were intraperitoneally injected into the mice model altered the expression of the genes, probably due to the oxidative stress and inflammation in the brains of the animals. The size, shape, type of the production, dose, and the route of exposure affect the nanotoxicity of the SNPs and their harmful effects on the rat organs.

There is not enough report on the influence of the biologically produced SNPs at their toxic and nontoxic doses in the rat brain, so we tried to produce the SNPs using the bacteria. The presence of SNPs was confirmed using different tests and their nontoxic doses were determined using MTT assay. At the final step, the nanoparticles were injected and their effects on the cerebellum of threats were analyzed using microscopic technique.

## 2 MATERIALS AND METHODS

### 2.1 Culture condition and production of silver nanoparticles

*Bacillus cereus* nanoparticles were purchased from the collection of industrial fungi and bacteria in Iran.

### 2.2 Synthesis of metal nanoparticles by extracellular method bacteria

Bacterial samples were first cultured on the Nutrient Broth medium in an incubator shaker at 150 rpm at 37°C for 24–48 h. After obtaining the biomass, the cellular biomass was separated from the culture medium by a millipour filter or by centrifugation at 1200 rpm for 10 min, and the supernatant or the liquid obtained from the filter was examined to produce metal nanoparticles. To test the production, the supernatants containing the tested microbes were mixed in erlens with 250 ml at a pH of about 5.8 with a concentration of 1 mm of silver nitrate solution in a final volume of 7.71% and placed in a shaker at 37°C for 24 h in the dark. Extracellular accumulation of metal particles will be investigated by changing the color of the culture medium. Controls used included incubation of culture medium alone and metal ions in the distilled water (Saifuddin et al., 2008).

### 2.3 Spectrophotometry (UV‐Vi)

In the next step, light absorption was examined by UV visible spectrophotometry from 2 to 700 nm. The highest absorption rate for the production of silver nanoparticles was expected at 10–450 nm (for each types of metal, the light absorption is different). The presence of a brown discoloration indicates the formation of silver nanoparticles in solution. This discoloration was due to (surface sprplasmon vibration) at the nanoparticle surface. The persistence of light absorption at a given wavelength over time indicated the dispersion of nanoparticles in the environment and their stability in the solution (Saifuddin et al., 2008).

### 2.4 Transmission electron microscope

To finally confirm the production of silver nanoparticles and measure the size of the particles produced, the samples obtained from each microorganism were examined by a transmission electron microscope (TEM). In this way, according to the instructions, the samples were placed on the grid and then they were photographed by a microscope at a voltage of 120 kV (Subramanian et al., 2010).

### 2.5 Assessment of the SNPs cytotoxicity in vitro

MTT testing is a common colorimetric method to evaluate the toxicity of substances on cell life, which is performed by measuring the regenerative power of tetrazolium dye (MTT)(3‐(4,5‐dimethylthiazol‐2‐yl)−2, 5‐diphenyltetrazolium bromide) by cellular enzymes and its conversion to formazan crystals with purple color. As a result of the reduction process, purple crystals of formazone color were formed. These crystals were then dissolved in a suitable solvent and quantified by spectrophotometric methods.

The amount of formazone crystal produced can indicate the percentage of living cells. The cell line used was fibroblast cells. First, the cells were inserted in specific concentrations (4 × 104) with 200 μl of DMEM (Dulbecco's Modified Eagle's medium) culture medium and 5% of bovine embryo serum in 96‐well plates. After 24 h of incubation in the cell culture incubator and reaching 80% of the accumulation, they were exposed to different concentrations of nanoparticles, which were reduced by half, respectively. Positive control was wells without nanoparticles. The plates were placed in a 25% CO incubator, which depends on the percentage of sodium bicarbonate in the culture medium for 24 h. The culture medium was then removed and 10 μl of 5 mg/ml MTT solution was added and stored at 37°C for 4 h. After separating the dye solution by the sampler, 100 μl of DMSO (dimethyl sulfoxide) was added to the wells and their light absorption at 550 nm was checked by ELISA reader. In this way, the highest concentration of nanoparticles that did not have cytotoxicity was determined.

According to the MTT test, which showed the IC50 in the second well, 1 ml of nanoparticles was first mixed with 3 ml of culture medium. Based on the fact that the rat has blood at 10% of its body weight, for rats weighing about 250 g, a nontoxic dose of 3–4 and a toxic dose of 6–8 ml were calculated and provided for injection.

### 2.6 Assessment of the SNPs toxicity in vivo

A total of 25 Wistar female rats weighing 200 ± 20 g were purchased from Pasteur Institute of Iran about 2 weeks prior to the commencement of the tests. The rats were kept under 12 h light−12 h dark cycles and were given water and food ad libitum and they did not receive any medicine. After 1 week of adaptation, Wistar female rats were randomly divided into five separate groups: group (A) as the control (*n* = 5) administered intraperitoneally with normal saline; group (B) (*n* = 5) administered intraperitoneally with the biological SNPs in the nontoxic dose; group (C) (*n* = 5) administered intraperitoneally with the nontoxic dose of the chemical SNPs; group D administrated with toxic dose of the biological SNPs; and group E administrated with toxic dose of the chemical SNPs. After 15 days, one rat from all groups underwent blood and tissue sampling to examine various maternal factors, and the rest of the female rats were ready to become pregnant. For impregnation, two male rats were used in each group. Pregnancy was confirmed after seeing the vaginal plaque. Due to the increase in the volume of injected nanoparticles, the injection was performed in four stages (two times in 2 consecutive weeks) by intraperitoneal method and in the last 2 weeks of pregnancy. Two weeks after the birth of rats and the end of infancy, neonates were sampled to increase the time of possible effect of nanoparticles on tissues. During pregnancy, the same diets were used in all groups, and after delivery, the cerebellar tissue of some newborns was sampled. For sampling, rats were first anesthetized by intraperitoneal injection of a combination of ketamine and xylazine, and then with a scalpel, the area of their cerebral cavity was cut off with scissors and forceps of cerebellar tissue and weighed. Then, samples were fixed using 10% formalin and after preparing histological sections. They were histologically evaluated using a light microscope. The results were analyzed by statistical tests.

## 3 RESULTS

### 3.1 Proving the SNPs formation

#### 3.1.1 Visible spectrophotometer

**FIGURE 1:**
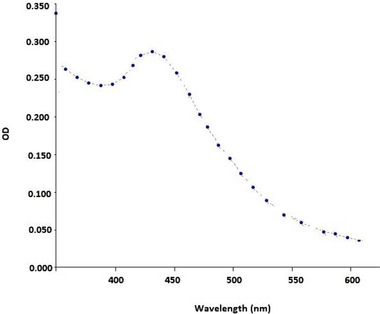
Visible spectrophotometer results of SNPs

In order to decrease the turbidity of the mixture, the mixture was diluted 1:15 using distilled water and light absorption was obtained according to Figure 1. Results indicated that SNPs solution had maximum absorbance peak around 429 nm.

#### 3.1.2 X‑ray diffraction analysis

**FIGURE 2:**
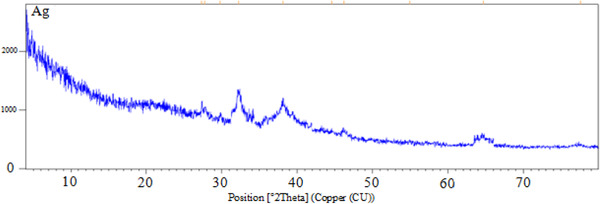
XRD results of SNPs production

The results of X‐ray diffraction (XRD) study showed the presence of peaks that belong to the elemental silver nanoparticles in the sample, which is a confirmation of the production of nanoparticles. According to the guide, the position of other peaks is also shown in Figure 2.

#### 3.1.3 Transmission electron microscope

**FIGURE 3:**
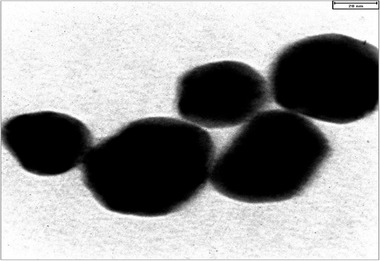
TEM micrographs that were obtained from the produced SNPs

The obtained SNPs were evaluated through TEM. The average sizes of SNPs were about 35 nm, with hexagonal and round shapes (Figure 3).

### 3.2 MTT assay

**FIGURE 4:**
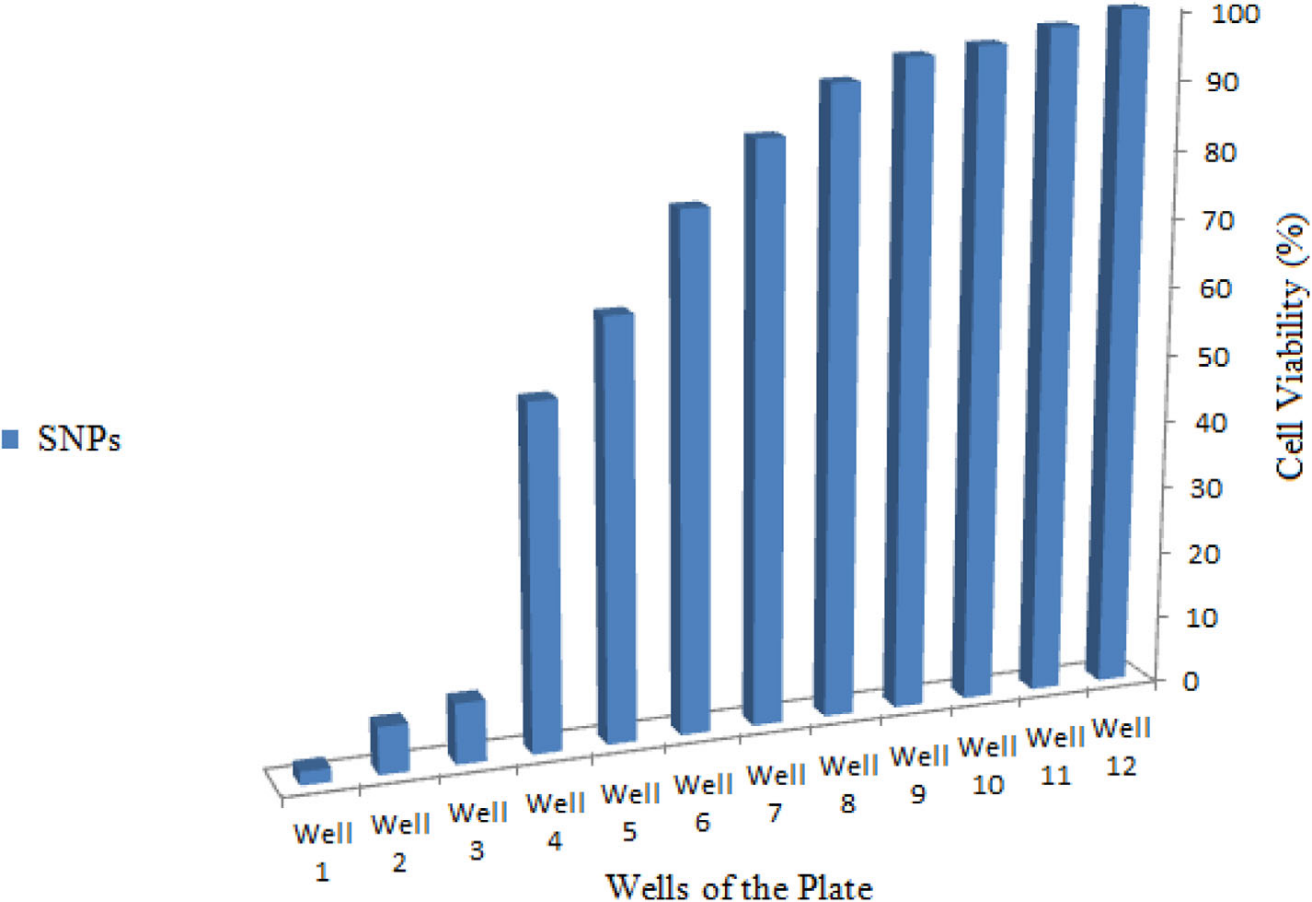
Percentage of cell viability obtained from spectrophotometer analysis by MTT method. Well 1 (0.25 mg/ml SNPs = 9.5 ppm/ml SNPs) had the highest concentration and well 11 (0.0002 mg/ml SNPs = 0.0093 ppm/ml SNPs) had the lowest concentration of SNPs. Well 12 was a control well without adding any SNPs

The results of the MTT test showed that well 4 is IC50. That means half the cells survived in that well. The concentration of the fifth well was determined as a nontoxic dose and the third well was determined as a toxic dose. The collection data were entered in Excel software and Figure 4 was drawn. Data were described with frequency percentage and diagram. Figure 4 shows the cell viability.

### 3.3 Histopathological results

#### 3.3.1 Control group

**FIGURE 5:**
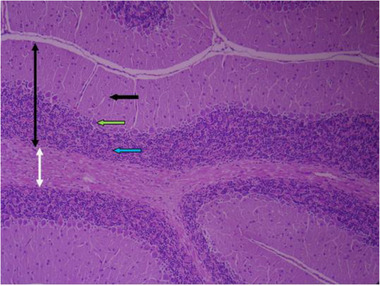
Rat cerebellar photomicrograp, control group× 100 (H&E staining)

**FIGURE 6:**
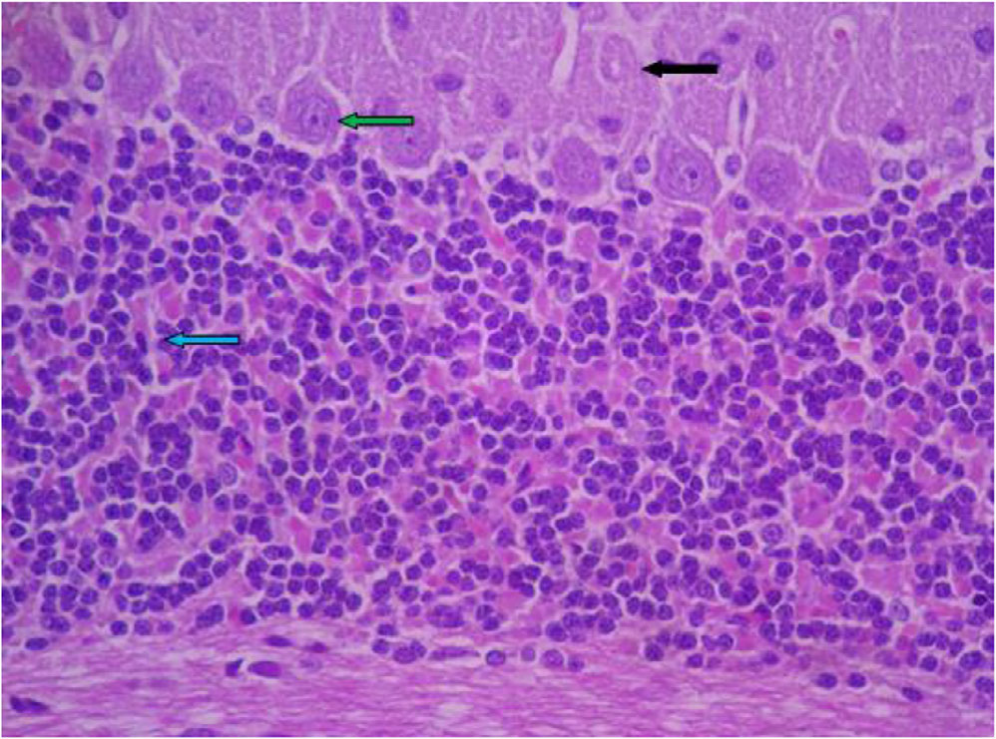
Rat cerebellar photomicrograp, control group× 400(H&E staining)

In the sections prepared from the control group, cerebellar tissue with natural characteristics and features along with tissue order and cohesion can be seen. The tree arrangement in the cerebellum of the control group is quite obvious and regular, and below the soft tissue of the gray matter (bilateral black arrow) and below the gray matter, the white matter (bilateral white arrow) is quite distinctive with normal characteristics. In the cerebellum, the gray matter that is on the surface consists of three layers. The first layer is just below the soft tissue and is called a molecular, which has a small cell with many nerve fibers (black arrow). The middle layer of the cell layer is called Purkinje, which consists of only one large, pear‐shaped cell row with two upper dendrites (green arrow). The cells of this layer are different in appearance and function from other cells in the cerebellum. The third layer of gray matter, called the granular layer, has about 10 rows of tiny nerve cells with dark nuclei that give it a grainy appearance (blue arrow). The white matter then lays beneath the granular layer, which lacks cellular bodies. In it, nerve fibers are seen along with neuroglial cells. Therefore, the characteristics of cerebellar tissue in the control group are completely normal (Figures 5 and 6).

#### 3.3.2 Nontoxic dose of chemical silver nanoparticles

**FIGURE 7:**
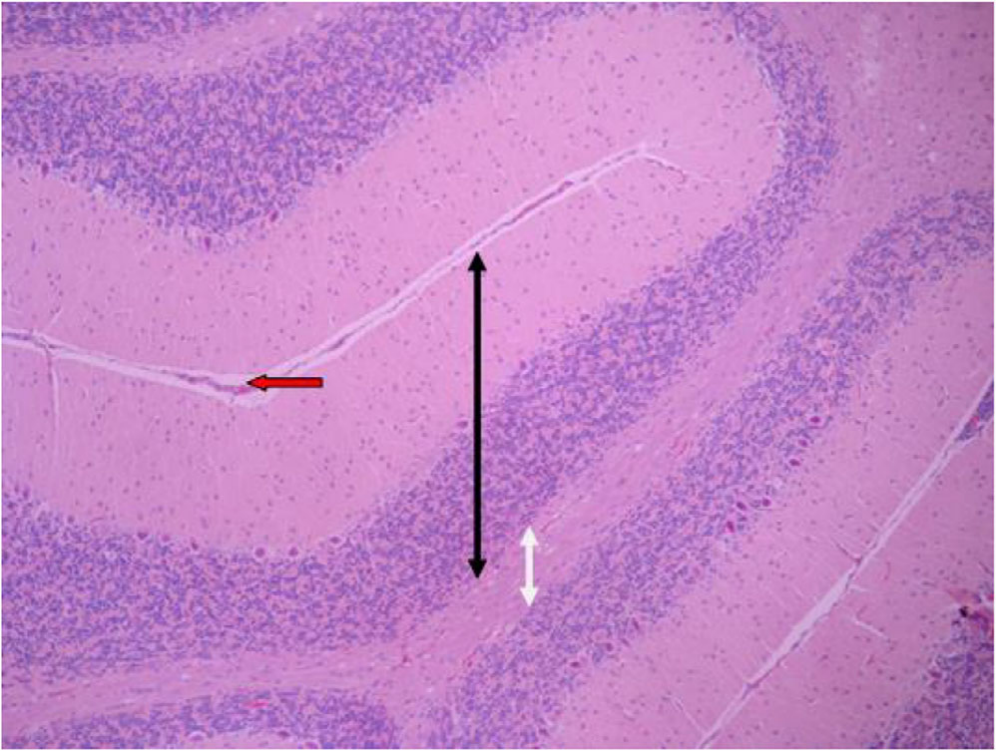
Rat cerebellar photomicrograp, nontoxic dose group of chemical silver nanoparticles× 100 (H&E staining)

**FIGURE 8:**
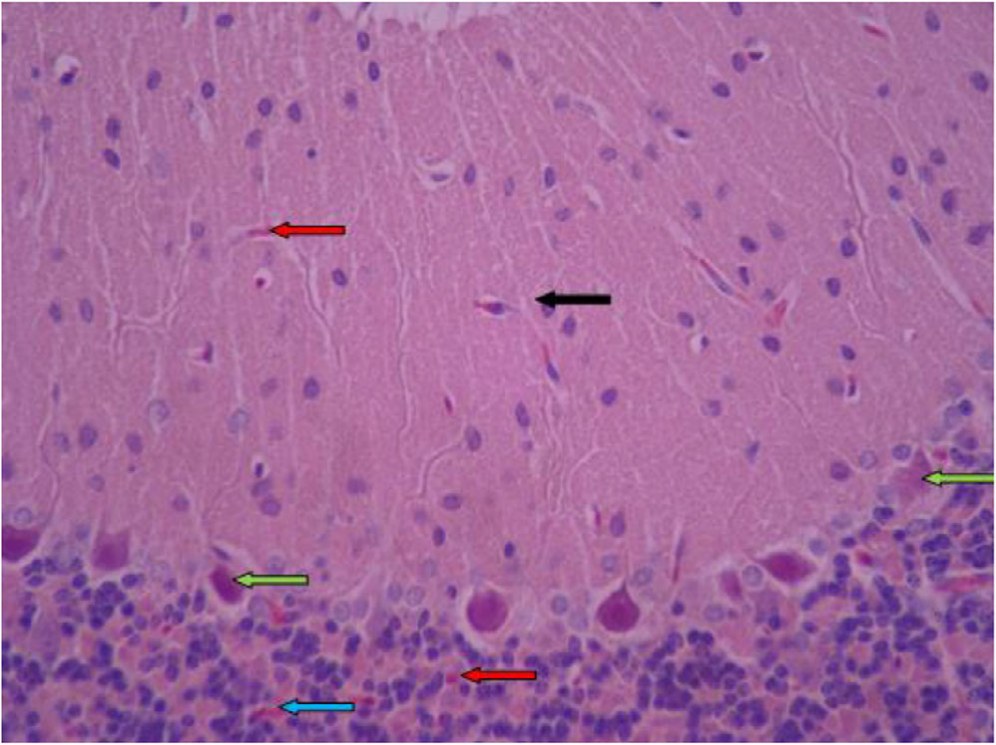
Rat cerebellar photomicrograp, nontoxic dose group of chemical silver nanoparticles× 400 (H&E staining)

In Figures 7 and 8, it is shown that in histological samples of the group receiving nontoxic dose of chemical silver nanoparticles, cerebellum tissue has regular tree arrangement with natural characteristics in soft, gray matter (bilateral black arrow) and white matter (bilateral white arrow). In some areas, such as the soft tissues, as well as inside the white and gray matter, congestion and vascular accumulation can be seen (red arrow). The molecular layer (black arrow) and the granular layer (blue arrow) have the right order and size. In the middle layer, most Purkinje cells have a good cell shape and size, but some of them have an inappropriate size and shrinkage with a dark cytoplasm and an unknown nucleus (green arrow).

#### 3.3.3 Nontoxic dose group of biological silver nanoparticles

**FIGURE 9:**
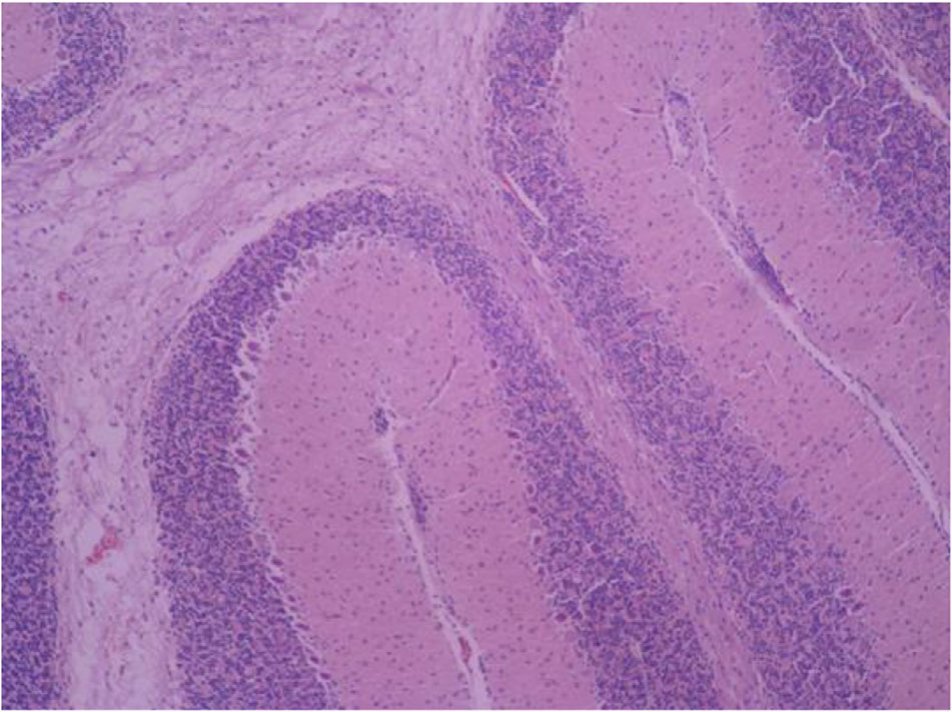
Rat cerebellar photomicrograp, nontoxic dose group of biological silver nanoparticles× 100 (H&E staining)

**FIGURE 10:**
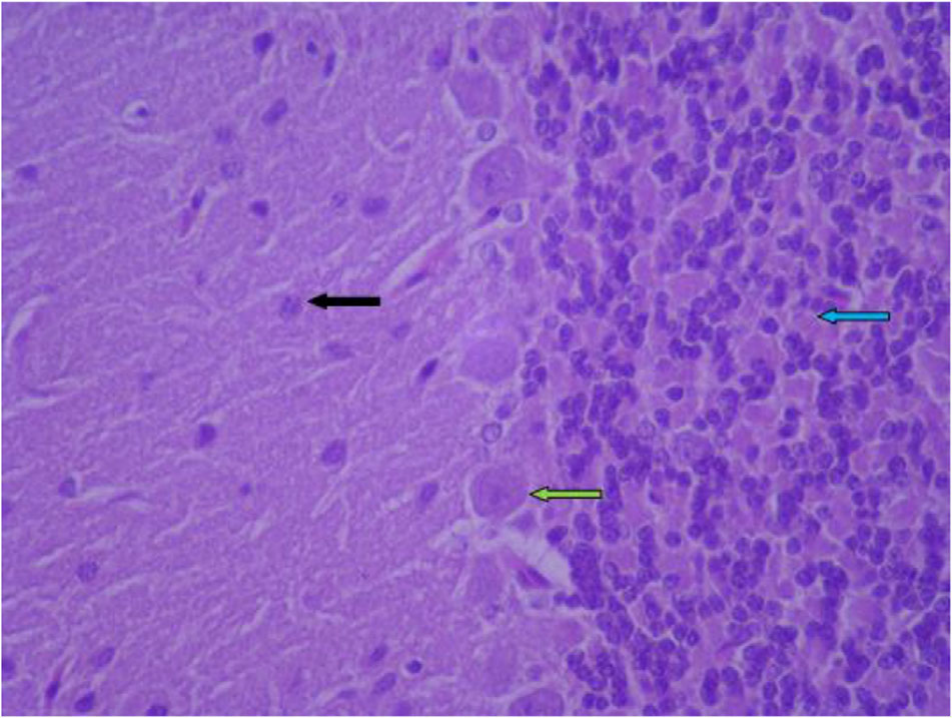
Rat cerebellar photomicrograp, nontoxic dose group of biological silver nanoparticles× 400 (H&E staining)

In cerebral tissue histological samples (Figures 9 and 10), the group receiving nontoxic dose of biological silver nanoparticles, gray matter (bilateral black arrow) and white matter (bilateral white arrow) with normal order and specifications and without any changes are visible. All layers of gray matter are clear and regular. In the molecular layer (black arrow), the size and texture characteristics are normal. Purkinje cells are seen with normal cell count and characteristics, clear nuclei, and regular cytoplasm (green arrow). The granular layer also has a normal cell size and number (blue arrow).

#### 3.3.4 Toxic dose group of chemical silver nanoparticles

**FIGURE 11:**
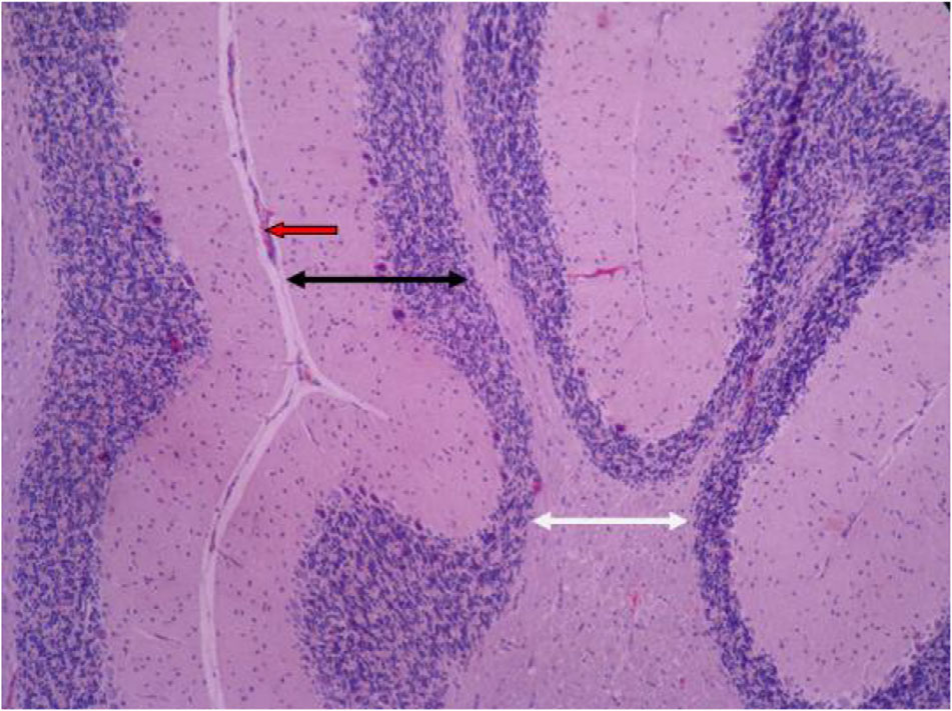
Rat cerebellar photomicrograp, toxic dose group of chemical silver nanoparticles× 100 (H&E staining)

**FIGURE 12:**
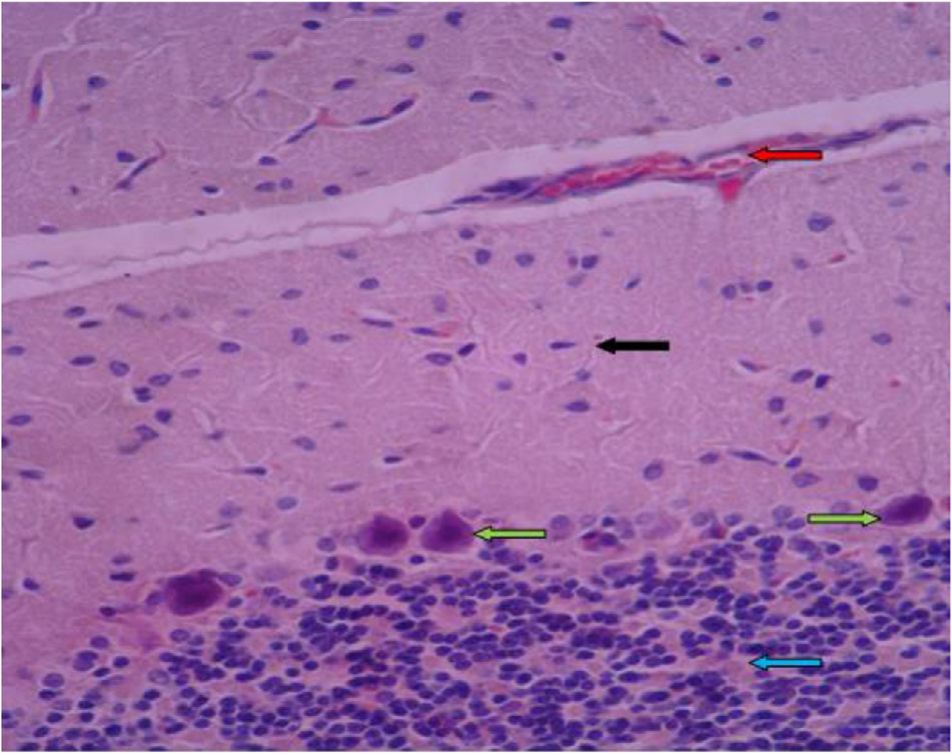
Rat cerebellar photomicrograp, toxic dose group of chemical silver nanoparticles× 400 (H&E staining)

In histological samples, the group receiving the toxic dose of chemical silver nanoparticles, cerebellum tissue has a regular tree arrangement and the characteristics of gray matter (bilateral black arrow) and white matter (bilateral white arrow) are completely normal. In some areas, such as the soft palate and inside the gray matter, vasodilation is seen with hyperemia (red arrow). In the molecular layer, the cell scattering is slightly irregular and high (black arrow) and in the granular layer, it is less in some areas of cell accumulation (blue arrow). In the middle layer, most Purkinje cells are associated with abnormal shapes, bold cytoplasm, and invisible nuclei, as well as reduced intercellular distances and reduced cell count (green arrow) (Figures 11 and 12).

#### 3.3.5 Toxic dose group of biological silver nanoparticles

**FIGURE 13:**
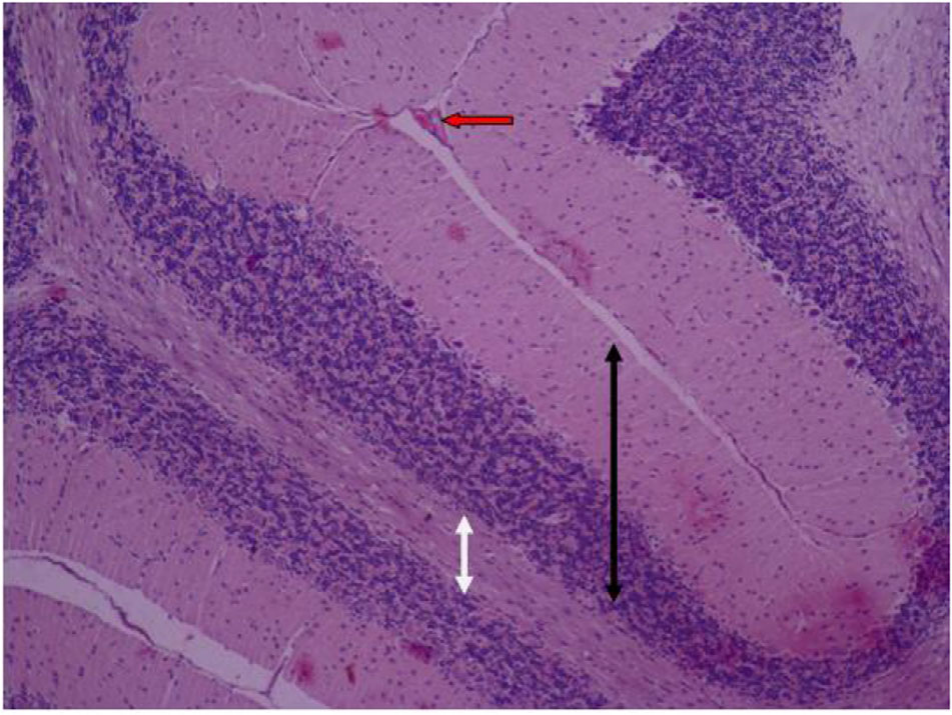
Rat cerebellar photomicrograp, toxic dose group of biological silver nanoparticles× 100 (H&E staining)

**FIGURE 14:**
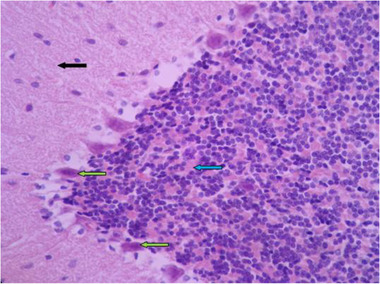
Rat cerebellar photomicrograp, toxic dose group of biological silver nanoparticles× 400 (H&E staining)

In histological samples (Figures 13 and 14), the group receiving the toxic dose of biological silver nanoparticles in cerebellum is normal and the characteristics of gray matter (bilateral black arrow) and white matter (bilateral white arrow) are normal. In some areas, vasodilation and hyperemia are observed (red arrow). Molecular layer (black arrow) and granular layer (blue arrow) with regular and integrated tissue and cellular characteristics are visible. In the middle layer, most Purkinje cells have normal cellular characteristics with appropriate cytoplasmic and nuclear characteristics, but the distances between them are irregularly incised, and some nuclei with long elongation and dark cytoplasm are seen (green arrow).

**FIGURE 15:**
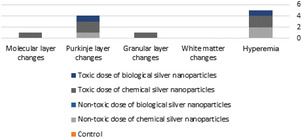
Classification of changes in the variables evaluated in cerebellar tissue

Based on the ANOVA results, in nontoxic chemical nanoparticles, cerebellum has a regular arrangement with natural characteristics in the soft tissue (Figure 15). In some areas, such as the soft tissues, as well as inside the white and gray matter, congestion and vascular accumulation can be seen. Purkinje cells are normal, but some are of an inappropriate size and shrinkage with an obscure dark and nucleated cytoplasm. Compared to chemical nanoparticles, in nontoxic biological nanoparticles, there are no changes in cerebellar cells.

In toxic doses of chemical nanoparticles in cerebellum cells, there is regular tree arrangement and the characteristics of gray and white matter are normal. The granular layer has less cell accumulation and in the middle layer, Purkinje cells with abnormal shape and reduced cell distance are seen. Nevertheless, in the toxic dose of biological nanoparticles, except irregular distances between Purkinje cells in the middle layer and some cells with high elongation and dark cytoplasm, there are no changes in cerebellar cells. Compared to toxic doses of chemical nanoparticles, these changes are insignificant.

## 4 DISCUSSION

The results of this study clearly showed the difference between the effect of nanoparticles produced biologically and chemically by showing that biological nanoparticles have less toxicity on cerebellar tissue cells compared to chemical nanoparticles and this toxicity in chemical nanoparticles is dose‐dependent.

One factor which influences the toxicity of the nanoparticles is the method of their production. As mentioned previously, there are three main types of nanoparticles production, which are named chemical, physical, and biological techniques. Literature reveals that most researches have focused on the chemical and physical methods of nanoparticles production, but there are a few studies on the in vivo toxic effects of the nanoparticles produced by biological techniques.

Biological method is regarded as the most suitable method for production because it is simple and fast and does not release any toxic by‐products into the environment. In this technique, some microorganisms are used for intra or extracellular production of nanoparticles. Although the exact mechanism of extracellular AgNPs nucleation is not clear, it is believed that some microbial external cellular proteins and other chemical functional groups of the carbohydrates are responsible in this process. These fine particles will be further coated by other exteracellular substances until AgNPs will be produced (Chowdhury et al., 2014; Osorio et al., 2021). By the use of the biological method, the produced nanoparticles usually have low toxic effects when they are used in vivo. Moreover, the production process can be easily scaled‐up (Siby & Beena, 2014).

It was also shown that biological gold nanoparticles also had low cytotoxic properties on liver tissue, which depends on the dose of nanoparticles (Yahyaei et al., 2019). In comparison, in the present research, biological silver nanoparticles in the toxic dose also made slight changes in the cerebellar tissue, are dose‐dependent, and did not cause any changes in the nontoxic dose. In another research, it was reported that in Embryonic period, silver nanoparticles have chemically destructive effects on cerebellar histogenesis at high and low doses. The nonbiologically produced SNPs are among the most frequently used nanoparticles that are reported as one of the toxic materials that may lead to harmful effects on human and ecosystem (Manuel et al., 2017). This type of nanoparticle is present in the drinking water, medical equipment, food products, clothes, and many other materials that may be inclose contact with the human body (Yahyaei et al., 2016). It has been reported that during pregnancy, the nonbiologically produced SNPs may enter the body and transfer from mother to her offspring. They can damage the cell membrane, DNA, and other important parts of the cell (Teng et al., 2021). In vitro analysis, it has been shown that exposure of the embryonic cells to the nonbiologically produced SNPs could up‐ and down regulate several genes. Tabatabaie et al. reported that administration of the nonbiologically produced SNPs during the pregnancy increased gene expression of monoamine oxidase and tyrosine hydroxylase, which are involved in the metabolism of dopamine in the brain of the rat offspring (Tabata, 2017). The nonbiologically produced SNPs can penetrate the body and accumulate in different organs, such as kidney, spleen, and liver. There are some reports on their crossing from the blood–brain, testis, and placental barriers (Yang et al., 2012). For example, there is a report about the inhalation of the nano‐sized materials and their penetration into the Central Nervous System (Söderstjerna et al., 2013). There are some reports about their toxicity in the rat offspring. It was shown that after intravenous and oral administration of the nonbiologically produced gold and silver nanoparticles, they could pass the blood–retinal, blood–brain, and placental barriers, accumulated in these organs and induced inflammation and oxidative stress in the experimental animal models. Wu et al. showed that maternal exposure to the nonbiologically produced SNPs during pregnancy induced effects on the hippocampal neurodevelopment due to the release of the silver ions. They reported that coating of the SNPs with polyvinyl pyrrolidine reduced the toxicity of the SNPs. Austinet al. have used histopathological examinations and showed that the nonbiologically produced SNPs induced some adverse effects on the development of the rat offspring (Austin et al., 2012). Although there are some researches about the nanotoxicity of the nonbiologically produced SNPs in vitro and in vivo (Wei et al., 2010), the toxicity of the SNPs produced by the safest method of nanoparticles production, the biological technique, was not fully understood (Kumari et al., 2012). There is no investigation about the transfer of the biologically produced nanoparticles through the placenta and BBB, which can be proved by the histopathological examinations.

In the present study, we also observed the destructive effects of chemical nanoparticles in toxic and nontoxic doses. In another research, chemical silver nanoparticles cause cerebellar damage and changes in Purkinje cells, which is consistent with our study. In another research (Pourali et al., 2019), they showed that the nonbiologically produced SNPs can penetrate the body and accumulate in different organs, such as kidney, spleen, and liver. The toxic effects of the nonbiologically produced SNPs may depend on the route of exposure, the dose, size, and shapes of the nanoparticles and the type of their production. They also showed that after maternal exposure to the biologically produced SNPs, they could pass through the placenta and induce mild alternations in the rat internal organs, which are consistent with our study.

Another study also showed that the biologically produced gold nanoparticles (GNPs) had the ability of conjugation with different types of chemotherapeutic drugs, but it is vital to choose the drugs which preserve their activity in the linking process (Yahyaei & Pourali, 2019). Yin et al. (2015) found that chemical silver nanoparticles cause mixed ataxia symptoms in rats, indicating impaired motor coordination and motor activity impairment. It was also revealed that nanoparticles can create a mixed gravitational degradation layer with a glial cell activating partner, which was consistent with the results of our study. They showed that maternal exposure to chemical silver nanoparticles could activate oxidative stress and apoptosis in the brain and affect and damage embryogenesis. Extensive apoptosis and oxidative stress were not identified in the results of our study. But tissue changes are quite evident, especially at doses of chemical nanoparticles.

In another study (Pourali & Yahyaei, 2015), AgNPs that were produced by bacterial culture supernatant had low and dose‐dependent cytotoxicity effects in the cell culture. Results have shown that the biologically produced AgNPs when used at their nontoxic doses had excellent wound‐healing properties in the animal model.

In the study of Pourali et al. (2016), both Gram positive and negative bacterial strains produced AgNPs due to the production of several types of enzymes, polypeptides, or polysaccharides that are responsible for the reduction of silver nitrate ions to AgNPs. The biologically produced AgNPs increased the epithelization, formation of the collagen bundles and fibroplasia, and decreased the angiogenesis and duration of completion of the epithelization when used in vivo.

## 5 CONCLUSION

The contrast with chemical nanoparticles and biochemical nanoparticles has less significant effect on the cerebellum cells.
